# The In Vivo Effect of Transcellular Permeation Enhancers on the Intestinal Permeability of Two Peptide Drugs Enalaprilat and Hexarelin

**DOI:** 10.3390/pharmaceutics12020099

**Published:** 2020-01-26

**Authors:** David Dahlgren, Markus Sjöblom, Mikael Hedeland, Hans Lennernäs

**Affiliations:** 1 Department of Pharmacy, Uppsala University, 751 23 Uppsala, Sweden; david.dahlgren@farmaci.uu.se; 2 Department of Neuroscience, Uppsala University, 751 23 Uppsala, Sweden; Markus.Sjoblom@neuro.uu.se; 3 Department of Medicinal Chemistry, Uppsala University, 751 23 Uppsala, Sweden; mikael.hedeland@ilk.uu.se; 4 National Veterinary Institute (SVA), 751 89 Uppsala, Sweden

**Keywords:** permeation enhancers, absorption-modifying excipients, oral peptide delivery, intestinal permeability, intestinal perfusion, pharmaceutical development

## Abstract

Permeation enhancers like sodium dodecyl sulfate (SDS) and caprate increase the intestinal permeability of small model peptide compounds, such as enalaprilat (349 Da). However, their effects remain to be investigated for larger low-permeability peptide drugs, such as hexarelin (887 Da). The objective of this single-pass perfusion study in rat was to investigate the effect of SDS at 5 mg/mL and of caprate administered at different luminal concentrations (5, 10, and 20 mg/mL) and pH (6.5 and 7.4). The small intestinal permeability of enalaprilat increased by 8- and 9-fold with SDS at 5 mg/mL and with caprate at 10 and 20 mg/mL but only at pH 7.4, where the free dissolved caprate concentration is higher than at pH 6.5 (5 vs. 2 mg/mL). Neither SDS nor caprate at any of the investigated luminal concentrations enhanced absorption of the larger peptide hexarelin. These results show that caprate requires doses above its saturation concentration (a reservoir suspension) to enhance absorption, most likely because dissolved caprate itself is rapidly absorbed. The absent effect on hexarelin may partly explain why the use of permeation enhancers for enabling oral peptide delivery has largely failed to evolve from in vitro evaluations into approved oral products. It is obvious that more innovative and effective drug delivery strategies are needed for this class of drugs.

## 1. Introduction

Oral administration is the preferred route of treatment for low molecular mass drugs for their ease of intake and high patient compliance [1]. The biotechnology-based pharma industries have tried to develop oral drug delivery systems for this administration route for these new modalities such as therapeutic peptides, proteins, antibodies, and genetic medicines [2]. Even so, peptide drugs are generally not orally administered because of their low intestinal luminal stability and/or permeability across the intestinal epithelial cells. The low intestinal permeability is related to their large size (Molecular mass > 800 Da) and low lipophilicity [3].

A thoroughly investigated strategy for oral peptide delivery is to reduce the integrity of the intestinal membrane barrier. This approach may be achieved with permeation enhancers (PE), also called absorption-modifying excipients, incorporated into the oral drug formulation [4]. As long as there are no concerns with luminal stability, a PE may increase the permeation of a peptide drug dissolved in the intestinal lumen [5]. This approach was recently FDA approved for semaglutide, a modified peptide containing 31 amino acids. Semaglutide has favorable properties for oral delivery: (i) high GI luminal stability, (ii) high potency, (iii) low clearance, and (iv) long terminal half-life. However, when administered orally with a PE (600 mg Sodium *N*-[8-(2-hydroxybenzoyl)amino] caprylate) in dog, its bioavailability increases but is still low (0.29%) and highly variable (CV = 200%), which illustrates the complexity of this drug delivery approach (data from [6]). 

The fatty acid caprate is a PE that has been investigated in a range of studies and clinical trials, and its approval as a food additive means that regulatory agencies view it as safe [7]. Several preclinical cell-monolayer studies claim that caprate regulates paracellular drug transport by affecting both sealing and pore-forming claudins in the tight junctions [8,9]. On the other hand, in vivo, it promotes absorption most likely because of its surfactant properties, where caprate incorporates into the lipoidal membrane, thereby increasing membrane fluidity and reducing epithelial integrity [8]. However, there is still some uncertainty if the in vivo effect of caprate is primarily related to the total intestinal dose or to the free dissolved concentration, which increases with luminal pH.

Recently, multiple mechanistic intestinal transport studies in rat have examined the effects of a range of PEs—including caprate and sodium dodecyl sulfate (SDS)—on intestinal permeation of a selected set of small model drugs (MM < 350 Da) [10,11,12]. SDS at 5 mg/mL in the intestinal lumen increases permeation in both absorptive and secretory directions in the rat single-pass intestinal perfusion (SPIP) model, while caprate at 2 and 5 mg/mL does not [13]. The effects of PEs are also substantially lower in the more in vivo relevant rat jejunal bolus model [11]. The lower effect in the jejunal bolus model could be due to multiple physiological and biopharmaceutical processes such as (i) luminal dilution, (ii) the rapid intestinal transit rate and subsequently short PE exposure time on the intestinal mucosa, and (iii) interaction with colloidal particles naturally present in the intestinal lumen [10,14]. Therefore, the physiological relevance of the rat SPIP model has also been evaluated, showing that the low enteric neural activity of the rat intestine following surgery can profoundly affect determinations of mucosal permeability following PE exposure [12]. However, these reports have only focused on the effect of PEs on the intestinal permeability of small model compounds. Larger molecules, such as peptides, have not been investigated. 

**Figure 1:**
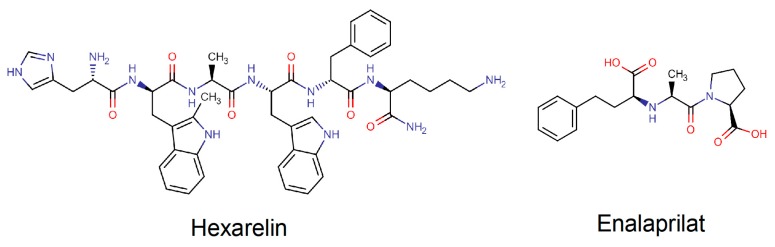
Molecular structures of two peptide drugs: hexarelin and enalaprilat.

The primary objective of this study was to investigate the absorption-enhancing effect of caprate and SDS during different luminal conditions for two peptide drugs, hexarelin (887 Da) and enalaprilat (348 Da); see Figure 1. The blood-to-lumen clearance was also measured for the clinical marker for mucosal barrier integrity, ^51^Chromium-labeled ethylenediaminetetraacetate (^51^Cr-EDTA). The second objective was to investigate if the absorption-enhancing effect of caprate for the two peptide drugs was due to the total free dissolved concentration or the total dose of caprate. SDS was included as a positive control in the study, as there is an abundance of data showings its permeation-enhancing effect as a surfactant in a range of preclinical models. These study objectives were evaluated by applying the in situ SPIP model in rat, which is considered an in vivo relevant model to investigate the effect of various agents on direct intestinal epithelial transport.

## 2. Materials and Methods

### 2.1. Active Pharmaceutical Ingredients, Pharmaceutical Excipients, and Other Chemicals

The molecular structures and some physicochemical properties of enalaprilat and hexarelin are presented in Figure 1 and Table 1. Enalaprilat, sodium caprate, hexarelin, pefabloc SC, SDS, bovine albumin, and thiobutabarbital sodium salt hydrate (Inactin) were purchased from Sigma-Aldrich (St. Louis, MO, USA). Sodium phosphate dibasic dihydrate (Na_2_HPO_4_·2H_2_O), potassium dihydrogen phosphate (KH_2_PO_4_), sodium hydroxide (NaOH), and sodium chloride (NaCl) were purchased from Merck KGaA (Darmstadt, Germany). ^51^Cr-EDTA was purchased from PerkinElmer Life Sciences (Boston, MA, USA).

### 2.2. Study Formulations

The intravenous isotonic (290 mOsm) solution contained 56 µM hexarelin and 63 µM enalaprilat. Six isotonic phosphate buffer perfusates (8 mM) were prepared, each containing 100 µM of enalaprilat and 90 µM hexarelin. The control solution contained no PEs, and five test formulations (two solutions and three suspensions) did contain PEs. Four of the test formulations were perfused at pH 7.4 and contained one of the following PEs: SDS at 5 mg/mL (17 mM) or caprate at 5, 10, and 20 mg/mL (25, 50, and 100 mM). At pH 7.4, SDS and caprate at 5 mg/mL were fully dissolved (solutions), while caprate at 10 and 20 mg/mL were above the solubility (suspensions). Caprate at 20 mg/mL was also investigated as a suspension at the physiologically relevant rat jejunal pH of 6.5, where its solubility is lower than at pH 7.4 (2 vs. 5 mg/mL). All perfusates contained the serine protease inhibitor, pefabloc SC at 0.3 mg/mL, because it completely inhibits the intestinal degradation of hexarelin in rat [15,16]. The PE concentrations of 5, 10, and 20 mg/mL correspond to oral doses of 1.0, 2.0, and 4.0 g administered with 200 mL of water [13]. 

The preparation of the perfusion formulations (100 mL) was done as described earlier [13]. There was no incompatibility, degradation, or apparent binding to glass/plastic of the study compounds in solution (pH 6.5, 37 °C) for 4 h. Osmolarity was determined after addition of all perfusate constituents (e.g., salt, PE, and water) by freezing-point depression using a Micro Osmometer (Model 3MO; Advanced Instruments, Needham Heights, MA, USA). 

### 2.3. Animals and Study Design

The surgical procedure and experimental setup of the rat SPIP experiment has been previously described [13]. The study was approved by the local ethics committee for animal research (No. C64/16—approval 18 November 2016) in Uppsala, Sweden. In short, male Wistar Han rats (strain 273) from Charles River Co. (Cologne, Germany), weight 270–363 g, were used. On the study day, the rats were anesthetized using an intraperitoneal injection of a 5% *w*/*v* thiobutabarbital solution (180 mg/kg). Body temperature was maintained at 37.5 ± 0.5 °C. To validate the condition of the animal, the systemic arterial blood pressure was continuously recorded by connecting the arterial catheter to a transducer operating a PowerLab system (AD Instruments, Hastings, UK).

One mL of the intravenous solution containing hexarelin (0.05 mg, 56 µM) and enalaprilat (0.21 mg, 63 µM) was administered as a bolus through the femoral vein, followed by a 0.5 mL-saline flush to ensure complete administration of the doses. 

At the SPIP experiment, the abdomen was opened along the midline and a jejunal segment of 10–15 cm was cannulated, covered with polyethylene wrap, and placed outside the abdomen. The bile duct was cannulated to avoid pancreaticobiliary secretion into the duodenum. After completion of surgery, ^51^Cr-EDTA was administered intravenously as a bolus of 75 µCi (0.4 mL), followed by a continuous infusion at a rate of 50 µCi per hour (1 mL/h) for the duration of the experiment. During the first 30 min following surgery, each intestinal segment was single-passed perfused with 37 °C phosphate buffered saline (6 mM, pH 6.5 or 7.4). This allowed cardiovascular, respiratory, and intestinal functions to stabilize and stable ^51^Cr-EDTA activity in blood plasma. The length of the intestinal segment was measured after the jejunal cannulation as was the wet tissue weight of the segment after the experiment. The perfusion rate was at all times 0.2 mL/min (peristaltic pump, Gilson Minipuls 3, Le Bel, France).

Each perfusate experiment was divided into two parts. In the first part, the segment was perfused with the control buffer solution (containing enalaprilat and hexarelin but no PE) for 60 min. In the second part, the segment was perfused for 75 min with one of the five test formulations (each containing enalaprilat and hexarelin, and one PE). SDS at pH 7.4 was the positive control, as this PE has been evaluated at 5 mg/mL in multiple rat SPIP studies [12,13,14]. Caprate at 5, 10, and 20 mg/mL were evaluated at pH 7.4, at which its solubility was 5 mg/mL. The 10 and 20 mg/mL concentrations (suspensions) were used to evaluate if concentrations above saturation had any impact on membrane permeability. Twenty mg/mL of caprate was also perfused at pH 6.5, at which its solubility is 2 mg/mL. This was to evaluate the impact of caprate dose vs. free caprate concentration on membrane permeability.

The five experiments were designed such that each rat was its own control. The experimental period started with a rapid filling (<30 s) of the whole segment with the perfusate (about 1.5 mL for a 10 cm segment). The intestinal segment and perfusates were kept at 37 °C, and all outgoing perfusate was collected and weighed at 15-min intervals. 

Blood samples of <0.3 mL were collected from the femoral artery for a maximum volume of 4 mL during each experiment. All sampled blood volumes were replaced by an equivalent volume of saline (0.9% NaCl) containing 70 mg/mL bovine serum albumin. For the intravenous administration, blood was sampled over 6 h (at 5, 10, 20, 40, 60, 90 min and at 2, 4, and 6 h). For the intestinal perfusion, blood was sampled at 15-min intervals for 135 min (7 samples). The blood samples were put on ice and centrifuged (5000× *g*, 3 min at 4 °C) within 10 min of sampling; 100 μL of the plasma was transferred to 0.5 mL micro tubes and stored at −80 °C until analysis.

### 2.4. Determination of Blood-to-Lumen Jejunal ^51^Cr-EDTA Clearance (CL_Cr-EDTA_)

Luminal perfusates and blood plasma at 0 and 135 min were analyzed for ^51^Cr activity (cpm) in a gamma counter (1282 Compugamma CS, Pharmacia AB, Uppsala, Sweden). A linear regression analysis of the plasma samples was made to obtain a corresponding plasma value for each perfusate sample. The ^51^Cr-EDTA clearance (*CL*_Cr-EDTA_) from blood-to-lumen was calculated using Equation (1) [17].

(1) $$CL_{Cr-EDTA}=\frac{C_{perfusate}\;\times \;Q_{in}}{C_{plasma}\;\times \;tissue\;weight}\times 100$$

where *C*_perfusate_ and *C*_plasma_ are the activities in the perfusate and plasma (cpm/mL) and where *Q*_in_ is the flow rate (mL/min). *CL*_Cr-EDTA_ was determined during the last 45 min for the control solution and during the last 60 min for the test solutions (the first 15 min in each period was for equilibration). The mean *CL*_Cr-EDTA_ value of the two perfusion periods was regarded as representative for each individual rat.

### 2.5. Jejunal Effective Permeability (P_eff_) Calculation

Jejunal lumen-to-blood effective permeability (*P_eff_*) of enalaprilat and hexarelin was determined based on a modification of the method described by Sjögren et al., 2015, which has been successfully implemented in human, dog, and rat [1,18,19,20,21]. In short, an input rate was acquired by deconvolution of the plasma concentration–time profiles following the intestinal perfusion. The intravenous pharmacokinetic data of hexarelin and enalaprilat from the two-compartment analysis was used as impulse response in the deconvolution. From the input rate, an absorption rate was then calculated by compensating for first-pass extraction (*F*_firstpass_) of each compound in the rat intestine and liver. The *F*_firstpass_ values for enalaprilat (0.99) and hexarelin (0.63) were based on literature data of the fraction of the model compound that is metabolized/excreted in the rat liver [22,23]. Plasma CL values were derived from the two-compartment analysis of the intravenous plasma data and an assumed rat liver blood flow of 47 mL/min/kg [24]. The *P*_eff_ (cm/s) was then calculated by relating the absorption rate to the intestinal luminal area using Equation (2):

(2) $$P_{eff}=\frac{absorption\;rate}{A\;\times \;C}$$

where *A* is the area of the exposed intestinal segment described as a smooth cylinder with a radius of 0.2 cm and *C* is the concentration entering the luminal segment. *P*_eff_ was evaluated from 0 to 135 min, and the mean *P*_eff_ value of the two perfusion periods (60-min control and 75-min test) was regarded as representative for each individual rat. 

### 2.6. Bioanalysis

Hexarelin and enalaprilat were quantified in plasma using ultrahigh performance liquid chromatography-tandem mass spectrometry (UHPLC-MS/MS). The plasma samples (20 µL) were transferred to a 96-well plate to which 50 µL of the internal standard for hexarelin (His* -D-Trp(2Me)-Ala*-Trp-D-Phe-Lys*-amide, where the asterisk (*) denotes ^13^C labels, 250 nM in water) and 100 µL of the internal standard for enalaprilat (enalaprilate-d5 100 nM in methanol) were added. The plate was vortex-mixed for 10 min (pulse 1200) after which it was centrifuged for 10 min at 2862× *g*. The supernatants were then injected into the UHPLC-MS/MS system. The column used was an Acquity UPLC Peptide BEH C18 (50 × 2.1 mm length × inner diameter, 1.7 µm particle size) from Waters Corp. (Milford, MA, USA). The mobile phase consisted of (A) 0.1% formic acid in water and (B) 0.1% formic acid in acetonitrile. Gradient elution was initially 2.0% B for 0.50 min, with a linear increase to 90% B for 1.5 min, held constant at 90% B for 0.30 min, then back to 2.0% B in 0.30 min, and constant again at 2.0% B for 0.40 min. The total run time was 3.0 min. 

The tandem mass spectrometer was a TQS micro from Waters Corp, and the ionization technique was positive electrospray. The quantification was performed in the Selective Reaction Monitoring mode (SRM) with the transitions 444.3 > 129 for hexarelin (collision energy 18 eV), 454.7 > 137 for hexarelin-IS (collision energy 20 eV), 349 > 206 for enalaprilat (collision energy 18 eV), and 354 > 211 for enalaprilate-d5 (collision energy 18). For quantification, calibrator samples were prepared by spiking standard solutions of the two analytes to blank plasma; the calibration curve was constructed by linear curve fitting using the chromatographic peak area ratio (analyte/internal standard) as a function of analyte concentration. The measurement intervals were 0.5–300 nM for hexarelin and 0.5–600 nM for enalaprilat. Representative chromatograms of enalaprilat and hexarelin and their internal standards are supplied as Supplementary Materials.

### 2.7. Statistical Analysis

The sample size in each study group was five or six rats on the basis of previous perfusion studies [12,13]. *P*_eff_ and *CL*_Cr-EDTA_ values, are expressed as the mean ± standard deviation (SD) or standard error of the mean (SEM). The *P*_eff_ and *CL*_Cr-EDTA_ ratio between the 45-min control and 60-min test period in the rat perfusion studies are also presented (Equation (3)):

(3) $$Ratio\;\left(CL_{Cr-EDTA}\;or\;P_{eff}\right)=\frac{mean\;value\;\left(test\;period\right)}{mean\;value\;\left(control\;period\right)}$$

The ratio was compared using the paired student’s t-test with the Benjamini–Hochberg multiple *t*-test correction. Multiple comparisons between groups were performed using a one-way ANOVA with a post hoc Tukey’s multiple comparison test. Log transformation of values was performed when the original measured data were heteroscedastic and not normally distributed; this was investigated using the Bartlett test. Differences were considered to be statistically significant when the *p*-value was smaller than 0.05.

## 3. Results

### 3.1. Plasma Profiles

**Figure 2:**
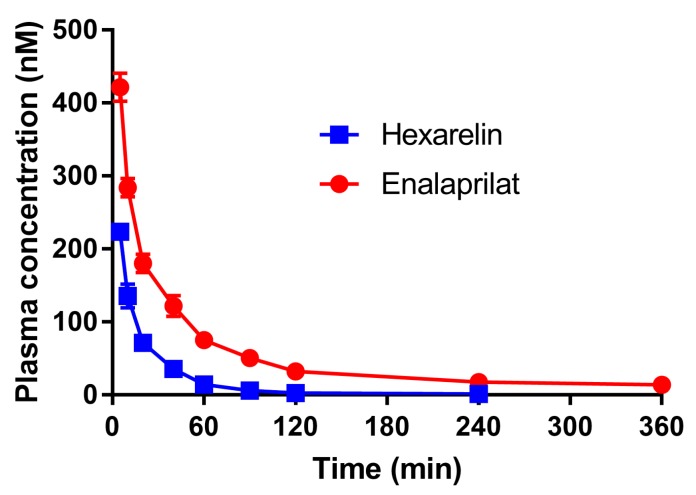
The mean (±SD) plasma concentration–time profiles (*n* = 2) following the intravenous bolus administration of 0.02 mg enalaprilat and 0.05 mg hexarelin.

The mean (±SD) plasma concentration–time profiles of enalaprilat and hexarelin following the intravenous administration are presented in Figure 2. The intravenous plasma concentration–time profiles of both enalaprilat and hexarelin best followed a two-compartment model.

The rat pharmacokinetic data from the two-compartment analysis of the intravenous plasma data (Figure 2) are presented in Table 2. 

**Figure 3:**
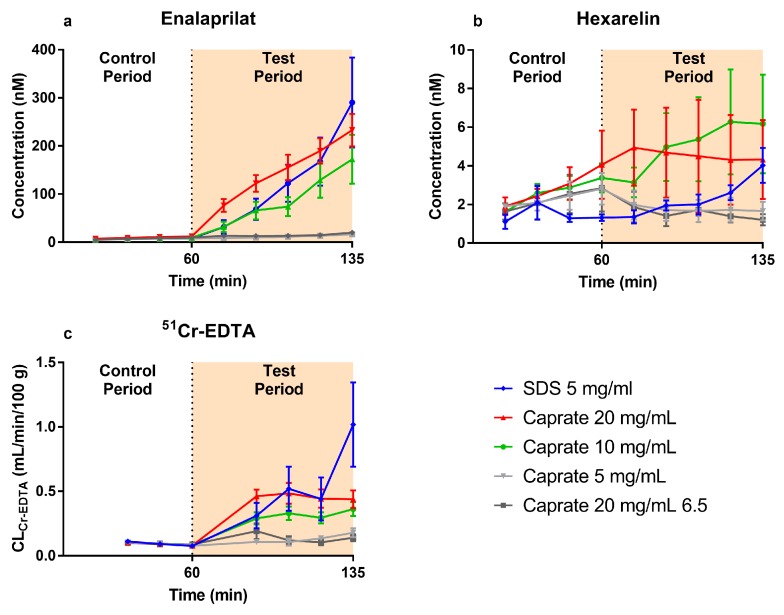
The mean (±SEM) plasma concentration–time profiles (*n* = 5/6) of (**a**) enalaprilat and (**b**) hexarelin and (**c**) the blood-to-lumen clearance of ^51^Cr-EDTA (*CL*_Cr-EDTA_), following the intestinal perfusions of a control solution for 60 min, followed by a 75-min perfusion of any of five test formulations containing a permeation enhancer: The control solution and all test formulations contained both 100 µM enalaprilat and 90 µM hexarelin. The perfusate pH was 7.4, and the permeation enhancers were sodium dodecyl sulfate (SDS) at 5 mg/mL and caprate at 5, 10, and 20 mg/mL. Caprate at 20 mg/mL was also tested at pH 6.5, where its solubility was 2 mg/mL instead of 5 mg/mL at pH 7.4.

The mean (±SEM) plasma concentration–time profiles of enalaprilat and hexarelin following the jejunal perfusions of the control solution (0–60 min) and five PE-containing test formulations (60–135 min) are presented in Figure 3a,b, respectively. These plasma data were used to determine *P*_eff_ and *P*_eff_ ratio values of enalaprilat and hexarelin using Equations (2) and (3).

There was a similar increase in plasma concentration–time profiles (test vs. control period) of enalaprilat induced by caprate at 10 and 20 mg/mL (pH 7.4, suspensions) and by SDS at 5 mg/mL (pH 7.4, solution). There was no increase in plasma concentration–time profiles (test vs. control period) of enalaprilat induced by caprate at 5 mg/mL (pH 7.4, solution) or by caprate at 20 mg/mL (pH 6.5, suspension).

There were only small differences in plasma concentration–time profiles in the control and test periods of hexarelin for all investigated caprate and SDS formulations. The only exception was caprate at pH 6.5 (20 mg/mL), where only 2/6 of the animals had plasma concentrations above the lower limit of quantification. This indicates that the permeability of hexarelin was lower at pH 6.5 compared to pH 7.4.

### 3.2. Blood-to-Lumen Jejunal ^51^Cr-EDTA Clearance Profiles

The mean (±SEM) *CL*_Cr-EDTA_ following the jejunal single-pass perfusions of the control solution (0–60 min) and five PE containing test formulations (60–135 min) are presented in Figure 3c. These *CL*_Cr-EDTA_ data were used to determine *CL*_Cr-EDTA_ ratio values using Equation (3). 

There was an increase in *CL*_Cr-EDTA_ in the test period induced by caprate at 10 and 20 mg/mL (pH 7.4, suspensions) and by SDS at 5 mg/mL (pH 7.4, solution). There was a slightly higher absolute *CL*_Cr-EDTA_ in the test period induced by caprate at 20 mg/mL compared to 10 mg/mL. There was no increase in *CL*_Cr-EDTA_ in the test period induced by caprate at 5 mg/mL (pH 7.4, solution) or by caprate at 20 mg/mL (pH 6.5, suspension), where the free caprate concentration is lower than at pH 7.4.

### 3.3. Lumen-to-Blood Effective Permeability (P_eff_) of Enalaprilat and Hexarelin 

**Figure 4:**
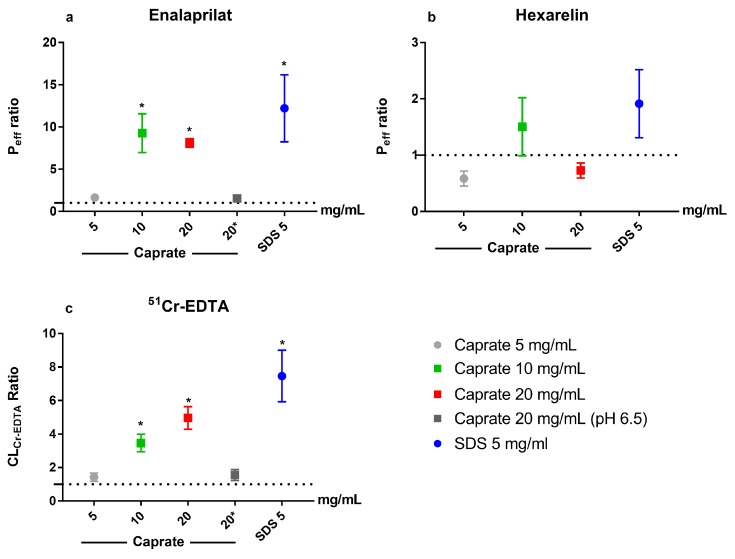
The mean ± SEM lumen-to-blood intestinal effective permeability (*P*_eff_) ratio of (**a**) enalaprilat and (**b**) hexarelin and (**c**) the blood-to-lumen ^51^Cr-EDTA clearance (*CL*_Cr-EDTA_) ratio, following intestinal perfusions of a control solution for 60 min, followed by a 75-min perfusion of five test formulations containing one permeation enhancer each: The perfusate pH was 7.4, and the permeation enhancers were sodium dodecyl sulfate (SDS) at 5 mg/mL and caprate at 5, 10, and 20 mg/mL. Caprate at 20 mg/mL was also tested at pH 6.5 (dark gray), at which its solubility was 2 mg/mL, instead of 5 mg/mL at pH 7.4. ■ The test formulation as a suspension; ● a solution; and * a significant increase in *P*_eff_ or *CL*_Cr-EDTA_ ratio.

The mean (±SD) effective permeability (*P*_eff_) was 7 times higher for enalaprilat (0.013 ± 0.009 × 10^−4^ cm/s) than hexarelin (0.0019 ± 0.0014 × 10^−4^ cm/s) in the first 60-min control period (*n* = 24). The enalaprilat and hexarelin *P*_eff_ ratio (calculated using Equation (3)) between the control and test period for the five test formulations are shown in Figure 4a,b, respectively. There was a significant increase in enalaprilat *P*_eff_ ratio (baseline vs. test) at pH 7.4 for caprate at 10 and 20 mg/mL (suspensions) and for SDS at 5 mg/mL (solution). Increasing the luminal caprate concentration from 10 to 20 mg/mL did not give any additional jejunal absorption. There was no increase in enalaprilat *P*_eff_ ratio for caprate at 5 mg/mL at pH 7.4 (solution) or for caprate at 20 mg/mL at pH 6.5 (suspension), where the free caprate concentration is lower than at pH 7.4. The *P*_eff_ ratio of hexarelin was unaffected by any of the five test formulations. The *P*_eff_ ratio of hexarelin for caprate at 20 mg/mL (pH 6.5) is not presented as there was only two rats with plasma hexarelin values over the lower limit of quantification, which suggests poor jejunal absorption and an absence of any effect of caprate at this luminal concentration and pH.

### 3.4. Blood-to-Lumen CL_Cr-EDTA_

The mean (±SD) *CL*_Cr-EDTA_ for the control solution (*n* = 24) was 0.092 ± 0.039 mL/min/100 g. The mean (±SEM) *CL*_Cr-EDTA_ ratio (calculated using Equation (3)) between the control and test period for the five test formulations is shown in Figure 4c. There was a significant increase in *CL*_Cr-EDTA_ ratio at pH 7.4 for caprate at 10 and 20 mg/mL (suspensions) and for SDS at 5 mg/mL (solution). There was no additional significant effect of increasing the caprate concentration from 10 to 20 mg/mL. There was no increase in *CL*_Cr-EDTA_ ratio for caprate at 5 mg/mL at pH 7.4 (solution) or for caprate at 20 mg/mL at pH 6.5 (suspension), where the free caprate concentration is lower than at pH 7.4.

## 4. Discussion

This rat single-pass intestinal perfusion (SPIP) study is part of a sequence of mechanistic studies evaluating the in vivo effect of permeation-enhancing (PE) excipients on intestinal transport of model drugs and marker compounds during various luminal conditions [11,12,13,14]. Here, we investigated the direct effects of two PEs (caprate and SDS) on the intestinal lumen-to-blood effective permeability (*P*_eff_) of two peptide drugs, enalaprilat and hexarelin, and the blood-to-lumen clearance of ^51^Cr-EDTA (*CL*_Cr-EDTA_). The absorption enhancement of caprate dose vs. dissolved free luminal active caprate concentration was also evaluated. 

Peptide drugs are generally not orally administered because of the low intestinal stability and/or poor permeability [25]. Luminal stability may be increased by modifying the peptide, for example, as has been done for the stable vasopressin analogue desmopressin, even if the absorbed fraction is below 1% [26]. Low permeability may, in turn, be circumvented by using PE pharmaceutical excipients in the drug delivery system; these increase the intestinal permeability by interacting with the mucosal membrane barrier [27,28,29]. However, there is only one approved oral drug product using PE technology to increase peptide absorption. This is in spite of the vast amount of experimental in vitro and in vivo investigations of this biopharmaceutical strategy [30,31,32]. A substantial number of these investigations have focused on the effect of PEs on small model drugs rather than large peptide-like drugs. Therefore, our investigation addressed the potential absorption-promoting effect of PEs on the intestinal permeability for a low permeable peptide in the lower molecular range (MM = 890 Da). These types of absorption studies in preclinical models provide useful knowledge regarding effects and mechanisms, which is considered necessary if permeation-enhancing drug-delivery technologies will ever reach a broad clinical breakthrough.

Our group has previously investigated the absorption enhancing effect of SDS, chitosan, n-acetylcysteine, and caprate in different absorption models [10,11,12,13,14]. In the SPIP model, chitosan and SDS increase the lumen-to-blood transport of four low permeation model compounds (acyclovir, atenolol, enalaprilat, and phenol red) as well as the blood-to-lumen clearance of ^51^Cr-EDTA (*CL*_Cr-EDTA_), a biologically inert, preclinical, and clinical marker for mucosal barrier integrity (molecular mass 345 g/mol, Log P −0.8) [33,34]. The relationship of PE-induced increase in blood-to-lumen *CL*_Cr-EDTA_ and lumen-to-blood transports is linear for of all four low permeation model compounds at all experimental conditions [12,13]. However, the relative increases in lumen-to-blood drug transport are different, i.e., the effect is inversely proportional to the basal permeability of the compounds. For instance, SDS at 5 mg/mL increases absorption 40-fold of the least permeable model drug enalaprilat (basal permeability: 0.011 × 10^−4^ cm/s) and 10-fold for the more permeable atenolol (0.13 × 10^−4^ cm/s) while not at all for the most permeable drug ketoprofen (1.6 × 10^−4^ cm/s) [13,35].

On the basis of these observations, we expected that the small intestinal absorption-enhancing effect of the PEs would be higher for hexarelin than enalaprilat at the optimized conditions applied in this study. Given that the basal permeability of hexarelin was >10-fold lower than enalaprilat (0.0019 ± 0.0013 × 10^−4^ cm/s vs. 0.013 ± 0.009 × 10^−4^ cm/s), it was surprising that none of the PEs at any luminal concentration enhanced absorption of hexarelin (Figure 2; Figure 3). At the same time, enalaprilat absorption increased greatly, in agreement with the increased *CL*_Cr-EDTA_ and previously reported data [12,13,14]. The absence of an effect of caprate and SDS on the permeability of hexarelin may be explained by ionic complexation between the cationic hexarelin and the ionic surfactants, which might reduce the free fraction of hexarelin in the intestinal lumen [36]. However, if there was an ionic complexation between hexarelin and caprate or SDS, a reduction in its membrane permeability should have been observed in the test period for caprate at 5 mg/mL and at 20 mg/mL (pH 6.5), as there were no membrane effects of these two formulations. In addition, there were no complexation effects observed between caprate and hexarelin in an in vitro study with the Caco-2 model. Instead, caprate increased the apparent permeability of hexarelin over 200-fold [16]. This substantially larger effect in Caco-2 is also in agreement with the previously reported higher effect of PEs in vitro compared to in vivo intestinal models [4].

It seems as if the transcellular permeation enhancers used in this in vivo study (SDS and caprate) were unable to fluidize the epithelial membrane sufficiently to accommodate any transcellular transport of hexarelin, whereas for enalaprilat, it was sufficient. This is regardless of the typically higher membrane permeation of at positively charged molecules (hexarelin at pH 6.5) compared to negatively changed ones (enalaprilat at pH 6.5) [37]. This can possibly be explained by molecular dynamics, where the thermodynamic cost of partitioning into and getting across the fluidized epithelial cell membrane is still too large for hexarelin [38]. The lack of an effect in this study may partly explain why the use of PEs for oral peptide delivery has failed to evolve into any clinical product. However, it is possible that a PE, such as chitosan or EDTA that act by increasing the paracellular permeability, would work for hexarelin [39]. This will be evaluated in an upcoming study.

Caprate (172 Da), a fatty acid with high intestinal permeability (human colonic *P*_eff_ = 1.4 ± 0.4 × 10^−4^ cm/s), has been proposed as a PE absorption-enhancing oral drug delivery systems [40]. It is a clinically interesting as it is approved as a food additive by the FDA without any upper dose limits [7]. Therefore, its use as an absorption enhancer has been investigated in a range of preclinical in vitro and in vivo models, and there is an abundance of data supporting its effect on the epithelial transport of low permeability model compounds [7]. Mechanistically, caprate has been proposed to increase paracellular permeability by modulating the phosphorylation and/or removal of tight junction-associated proteins [9,41]. However, recently, it is more accepted that its absorption-enhancing mechanism is primarily related to its membrane-perturbing effects as a surfactant [8]. For instance, there is a relationship between its concentration, absorption enhancing effect, and histological damage [42,43]. As a surfactant, its incorporation into the lipoidal membrane increases the membrane fluidity, thereby increasing transcellular permeability. 

In the rat SPIP model, the effective caprate concentrations are between 4–20 mg/mL [7]. However, this is higher than its solubility (2 mg/mL) at the physiologic jejunal pH of 6.5 [13]. Accordingly, in a previous rat jejunal SPIP study, we did not observe any absorption-enhancing effect on small low permeability model compounds of caprate at 2 mg/mL [13]. Further, the effect of 4 mg/mL caprate was increased in a rectal perfusion study when the pH was increased from 6.5 to 7.4, which is most likely explained by the higher caprate solubility (5 mg/mL) at the higher pH [40]. However, in an intraintestinal rat bolus study, a caprate dose of 11.4 mg/mL had no effect, regardless of formulation pH (6.5 and 7.4) [11]. This is likely explained by the very rapid ability of the intestinal lumen to normalize pH, which results in a decreased free caprate concentration [20].

These inconsistent results motivated us to further evaluate caprate in the rat SPIP model at even higher concentrations. In this study, we single-pass perfused caprate at 5, 10, and 20 mg/mL at pH 7.4, where its solubility was 5 mg/mL, and at 20 mg/mL at pH 6.5, where its solubility was 2 mg/mL. At pH 7.4, the permeability of enalaprilat increased 9-fold for caprate at both 10 and 20 mg/mL but there was no effect of the 5 mg/mL. This shows that a saturated caprate solution (5 mg/mL) is not sufficient for an effect; a luminal reservoir is also needed. The reservoir most likely replenished any absorbed caprate that was transported away from the perfused segment, thereby prolonging the saturated concentration. Further support for this reservoir hypothesis is that there was no difference in the effect of the 10 and 20 mg/mL concentrations, which are two and four times higher than the aqueous solubility of caprate, respectively. The lack of an effect of caprate at 5 mg/mL indicates that its rapid intestinal absorption results in an effective concentration only being upheld in a small part of the perfused segment. It is also evident that the free dissolved concentration is the driving force for the effect of caprate rather than the total dose, as there was no effect of 20 mg/mL caprate at pH 6.5, where its solubility is 2 mg/mL. In contrast to caprate, there are no pH-solubility issues with SDS, the positive-control surfactant used in this study. Accordingly, the effect of SDS at 5 mg/mL on the permeability of enalaprilat and *CL*_Cr-EDTA_ was unaffected by pH (6.5 vs. 7.4) [13]. 

These results show that an in vivo effect of caprate requires high oral doses in combination with some pharmaceutical component that can uphold a local pH adjacent to the epithelial membrane at about 7.4. Any permeation-enhancing clinical drug-delivery system relying on caprate will consequently need take dose, solubility, and local conditions (pH) into consideration. These results also highlight that any preclinical model that does not take blood flow into consideration will have a limited clinical relevance for the evaluation of PEs that are absorbed over the mucosal membrane, as the extensive mesenteric blood flow creates constant sink conditions.

In conclusion, this rat SPIP study showed a PE-induced increase in small intestinal permeability for two small (<348 Da) low-permeability model compounds: enalaprilat (absorptive) and ^51^Cr-EDTA (excretion). However, there was no absorption-promoting effect for either of these two PEs at any of the investigated luminal concentrations for the larger model peptide hexarelin (887 Da). This difference may explain why PEs for enabling oral peptide delivery have largely failed to evolve from in vitro evaluation into any clinical product. It is obvious that more innovative and effective drug delivery strategies need to be developed for this class of drugs. Further, the permeation-enhancing effect of caprate on enalaprilat was only at high luminal concentrations (from 10 mg/mL) and only at pH 7.4, where the free caprate concentration is above 5 mg/mL. Caprate requires a high free dissolved concentration, which means that the luminal pH must be raised from 6.5 to 7.4. The caprate dose must also be above its aqueous saturation concentration because a luminal reservoir is needed to replenish any caprate absorbed in the intestinal segment. Consequently, any clinical drug-delivery system relying on caprate for permeation enhancement must take both its dose and solubility as well as local pH conditions into consideration. 

## Figures and Tables

**Table 1:** Some physicochemical properties of the biopharmaceutics classification system (BCS) class of enalaprilat and hexarelin.

Drug (BCS Class)	MM (Da)	pK_a_	PSA	HBA/HBD	Log P	Log D_7.4_	Log D_6.5_
Enalaprilat (III)	348	3.17 ^b^/7.84 ^a^	102.1	6/3	−0.13	−1.0	−1.0
Hexarelin (III)	887	-	300	9/11	0.73	−2.26	−3.40

^a^ acid, ^b^ base, HBA/HBD—hydrogen bond acceptor/donor, Log D_7.4/6.5_—*n*-octanol−water partition coefficient at pH 7.4/6.5, Log P—*n*-octanol−water coefficient, MM—molar mass, pK_a_—dissociation constant, PSA—polar surface area.

**Table 2:** Mean (*n* = 2) pharmacokinetic data from the two-compartment analysis of the intravenous rat (340 g) plasma concentration-time profiles (Figure 2), including clearance (CL), volume of distribution at steady state (Vss), and both intercepts (C_1_ and C_2_) and slopes (λ_1_ and λ_2_) of the first and second terms: Individual values of rat 1 and 2 in parenthesis.

Drug	CL (L/h)	Vss (L)	C_1_ (nM)	C_2_ (nM)	λ_1_ (h^−1^)	λ_2_ (h^−1^)
Enalaprilat	0.18 (0.18/0.17)	0.34 (0.30/0.37)	428.6 (449.1/408.0)	87.7 (105.5/70.0)	3.9 (4.6/3.3)	0.38 (0.45/0.31)
Hexarelin	0.62 (0.62/0.61)	0.22 (0.21/0.24)	355.2 (416.9/293.5)	133.7 (149.1/118.3)	13.7 (17.5/10.0)	2.08 (2.26/1.91)

## Supplementary Materials

The following are available online at https://www.mdpi.com/1999-4923/12/2/99/s1, representative chromatograms of enalaprilat and hexarelin and their internal standards.

## Abbreviations

AME	absorption-modifying excipient
*CL*_Cr-EDTA_	clearance of ^51^Cr-EDTA
*P*_eff_	intestinal effective permeability
PE	permeation enhancer
SDS	sodium dodecyl sulfate
SPIP	single-pass intestinal perfusion
